# Supplementary material for: Ensemble optimal interpolation for adjoint-free biogeochemical data assimilation
Source: PLoS One. 2023 Sep 5;18(9):e0291039. doi: 10.1371/journal.pone.0291039 (PMC10479889; doi:10.1371/journal.pone.0291039)
Supplement: S1 File — (PDF) [file pone.0291039.s001.pdf]

Supporting Information for *Ensemble optimal interpolation for adjoint-free biogeochemical data assimilation*

S1 Splitting the computation

One benefit of the computation of the EnOI increment, as in Eq. (1), is that it can be efficiently subdivided for parallelization and to reduce computer memory usage without loss of accuracy. While more sophisticated algorithms exist Anderson and Collins (2007); Sakov et al. (2012), a simple way to achieve this division is to split the model state into small segments, such as the part of model state associated with each individual variable or, to create even smaller segments, subsets thereof. Generally, we can split the computation of the 4dEnOI increment $\mathbf{s} = \mathbf{x}^* - \mathbf{x}$ into n_d parts by defining $n_\mathrm{d} + 1$ integer indices

$$1 = i_1 < i_2 < \ldots < i_{n_\mathrm{d}+1} = n_\mathrm{state} + 1. \tag{S1}$$

The increment can then be computed for each part according to

$$\mathbf{s}[i_k : i_{k+1}] = (\alpha\, \mathbf{L}[i_k : i_{k+1}, :] \circ \mathrm{cov}\,(\mathbf{X}[i_k : i_{k+1}, :], H\mathbf{X}))\, \mathbf{k}_{2d}, \tag{S2}$$

where square brackets denote indexing, row and column indices are separated by commas, the $i_k : i_{k+1}$ notation denotes indexing from i_k to i_{k+1}, not including i_{k+1}, and : by itself indexes the entire row or column. The vector $\mathbf{k}_{2d} = \left(\alpha\, \mathbf{L}' \circ \mathbf{HBH}^\mathrm{T} + \mathbf{R}\right)^{-1} (\mathbf{y} - H\mathbf{x})$ can be precomputed and remains constant for each part.

Implementation of the approach in Eq. (S2) requires only reading in a subset, or slice, of the model state for the entire ensemble and computing the localization weights for the associated subset of grid points. It is straightforward to parallelize this procedure, as the computations for one slice do not require knowledge of other slices. The size of the matrices involved in the computation shrinks to $(x_{i_{k+1}} - x_{i_k}) \times n_\mathrm{ens}$ or $n_\mathrm{ens} \times n_\mathrm{obs}$, where $(x_{i_{k+1}} - x_{i_k}) << n_\mathrm{state}$ for each slice, reducing the memory requirement significantly based on the choice of the slicing indices in typical applications where $n_\mathrm{obs} < n_\mathrm{state}$. As an example, in our implementation with $n_\mathrm{obs} = 29\,000$ observations in a typical 4-day data assimilation cycle, and a state size of $n_\mathrm{state} = 7\,419\,609$ (not counting model grid cells on land), computing $\mathrm{cov}\,(\mathbf{X}, H\mathbf{X})$, would require calculating a $7\,419\,609 \times 29\,000$ covariance matrix with more than $2 \cdot 10^{11}$ elements, exceeding the memory available on our cluster computer. Slicing the domain in the north-south direction and treating each model variable individually leads to a maximum matrix size of $7\,602 \times 29\,000$ (the size is variable-dependent: for example, sea level anomaly is a 2-dimensional variable, different in size from 3-dimensional variables), which can easily be increased or reduced based on the computational resources available.

Here, we did not examine an additional step to improve the computational efficiency of the DA update step, which is referred to as "local analysis" (Sakov and Bertino 2011) or "domain localization" (Carrassi et al. 2018): By applying a threshold to the localization weights and setting entries in $\mathbf{L}$ that are below the threshold to zero, spatially distant model state and observation locations can be ignored in the computation of $\mathbf{BH}^\mathrm{T}$. For example, when processing a slice of the model state

associated with a given horizontal coordinate, spatially distant observations, whose localization weights are zero for the whole slice, can be ignored, setting entries in $\mathbf{s}[i_k : i_{k+1}]$ to zero, without even computing the covariance term in Eq. (S2).

References

Jeffrey L. Anderson and Nancy Collins. Scalable implementations of ensemble filter algorithms for data assimilation. *Journal of Atmospheric and Oceanic Technology*, 24 (8):1452–1463, 2007. doi: 10.1175/JTECH2049.1.

Alberto Carrassi, Marc Bocquet, Laurent Bertino, and Geir Evensen. Data assimilation in the geosciences: An overview of methods, issues, and perspectives. *Wiley Interdisciplinary Reviews: Climate Change*, 9(5):1–50, 2018. doi: 10.1002/wcc.535.

P. Sakov, F. Counillon, L. Bertino, K. A. Lister, Peter R. Oke, and A. Korablev. TOPAZ4: An ocean-sea ice data assimilation system for the North Atlantic and Arctic. *Ocean Science*, 8(4):633–656, 2012. doi: 10.5194/os-8-633-2012.

Pavel Sakov and Laurent Bertino. Relation between two common localisation methods for the EnKF. *Computational Geosciences*, 15(2):225–237, 2011. doi: 10.1007/s10596-010-9202-6.
