# Supplementary material for: Ensemble optimal interpolation for adjoint-free biogeochemical data assimilation
Source: PLoS One. 2023 Sep 5;18(9):e0291039. doi: 10.1371/journal.pone.0291039 (PMC10479889; doi:10.1371/journal.pone.0291039)
Supplement: Attachment — Submitted filename: Review_Mattern-Edwards_Plos1.pdf [file pone.0291039.s002.pdf]

ENSEMBLE OPTIMAL INTERPOLATION FOR ADJOINT-FREE BIOGEOCHEMICAL DATA ASSIMILATION

J.P. MATTERN, C.A. EDWARDS

In this paper, the authors present a variant of 4dEnOI with application to a coupled ocean-biogeochemical model. Compared to traditionnal EnOI, they suggest to run an ensemble in order to provide flow-dependent statistics. The main differences with ensemble-based Kalman filter and smoother is that only one member is updated, while the remain part of the ensemble evolves freely accordingly to the dynamics of each member. A strategy of regeneration of the ensemble is suggested when the observation-aware member tends to be steered awy from the ensemble. In this case, it imples the computation of the most dominant principal components of the forecast anomaly matrix to resample the distribution. Furthermore, the authors suggest a strategy of variable localization, introducing unknown hyperparameters that aim to weaken cross-corellation among variables. Numerical experiments are done with a coupled model encompassing the domain of the California Current System developped at UCSC. The biogeochemical model is a simple NPZD model coupled with ROMS and real observations are assimilated (satellite and in situ data). Finally, the authors compared the performances of several variants of their 4dEnOI with a 4dVar using a log-transformation.

The manuscript is well written and the topics is relevant, beacuse building data assimilation (DA) systems for coupled ocean-biogeochemical models remains challenging. However, this work has some weaknesses:

- The diagnostics shown in the numerical experiments are computed with the assimilated observations. Unfortunately, we can not conclude on the benefits of the DA system only from what ones observes, because improving the misfit to assimilated observations does not imply an improvement of the unobserved variables. This is particularly true in the framework of ocean biogeochemistry (impact on nutrient concentrations?). I think that the authors have to compare to "independant" observations. Why not using the in situ data for the diagnostics, and so not assimilaing them? Evolution of the nutrient concentrations could also be shown. I am sorry, but just comparing the decreases in the observation term of the 4dVar cost function is not enough.
- The 4dEnOI with variable localization, as suggested by the authors, is based in questionnable choices. The first one is to run an ensemble in the forecast step, but updating only one member. In a lot of operational ensemble-based DA systems, the most expensive task is to run the ensemble during the forecast step, not to apply the analysis step. Considering that a regeneration of the ensemble may be required, I am not sure that the benefits of the 4dEnOI are that large. Unfortunately, there is no comparisons to an EnKF nor an EnOI in the numerical experiments. The introduction of the variable localization is very questionnable, and seems to be introduced to mitigate errors in the estimation of the forecast error covariance matrix. Because it introduces unknown hyperparameters (no strategies are introduced to estimate them), and good performances are obtain with low correlation among the biogeochemical variables (monovariate analysis scheme?), I really need a better intercomparison among the state-of-the-art algorithms to start to be convinced.

- I am surprised that no comparisons are made against ensemble-based method like an EnKF, an ensemble smoother, a hybrid EnOI or a static 4dEnOI. Regarding the EnKF and the hybrid EnOI, they can assimilate asynchronous observations. So I wonder why the authors did chose a 4dVar with a possible static **B**. The authors should explain why they did not choose at least one of these method to highlight the benefits of their approach.

First of all, I apologize to the authors for the delay of my report. However, to my point this manuscript is not ready for publication. Depending on the time the authors have to provide a revised version of the manuscript, I would suggest a major revision or a rejection (with an encouragement to resubmit the new version of the manuscript). More details are provided in the following list of comments.

Comments.

- Abstract: "our 4dEnOI reference implementation performs similarly well to the 4dVar benchmark in lowering the observation misfit": I wonder why the authors do not compare the performances of their approach with data that have not been assimilated. Improving the misfit to the observations that are assimilated is the minimum that ones expects with a data assimilation system.
- p.1, l.4-6 "DA is applied to coupled physical-biogeochemical ocean models, where it yields large improvments in the model's state estimates (Edward et al. 2015, Mattern et al. 2015)": The authors refer only to their previous works while this topic has been investigated by many other groups. I would suggest to add references.
- p.1, l.9-15 "Variational DA techniques, one group of widely-used DA techniques [$\cdots$] Ensemble-based DA techniques, the second group of widely used DA techniques, rely on statistics [$\cdots$] estimates": This splitting into two groups is not realy relevant now, because of the development en ensemble-based varational approaches (hybrid **B** matrix, EnVAr, Iterative EnKS, 4DEnvar, etc..). I would rather reword with one group relying on optimal control theory (4DVar) and the second one from the linear estimation theory (Kalman filter).
- p.2, l.67 - p.3, l.74 "Instead, it is based on flow-dependant statistics which are obtained from a dynamical ensemble [$\cdots$] making it suitable for complex biogeochemical models with many variables and consequently a very high-dimensional state vector." The authors suggest to dynamically run an ensemble to estimate the forecast mean and error covariance matrix within the DA process as done with an Ensemble Kalman smoother. However, they suggest to update only one member, and not the full ensemble. I am not sure to understand the rational of such approach. Usually, it is the forecast step that is the most expensive in terms of computational cost and time in an ensemble-based DA system for coupled models in high dimension. The analysis step remains cheaper compared to the forecast step. So having the ensemble, I do not think it changes that much the total computational cost and time to update one member rather than the full ensemble, and it would allow the estimation of the analysis error covariance matrix. Could the authors comment on my remark ?
- p.3, l.80 "the EnOI implementation to a 4dVAR-based DA system": The comparison to a 4dVar algorithm is interesting. However, this system does not use a flow-dependant error covariance matrix. I know that it is difficult to assess the performances of their 4dEnOI against all the existing algorithms, but could the authors better justify why they did not compare their results with a standard 4dEnOI or an ensemble-based Kalman smoother?
- p.4,l.119-126 "Unlike many other ensemble-based DA techniques like the EnKF, the remaining ensemble is not updated with data directly, but run alongside the first ensemble member [$\cdots$] passively updating the full ensemble with observations": With the update of only one member, it is not posible to reduce the

uncertainty in the solution through the estimation of the analysis error covariance matrix. Furthermore, it raises issues in the dynamics of the member wich is updated with the observations, and the other members of the ensemble. The authors suggested to regenerate the ensemble to remedy for these issues. However, it requires to compute the SVD of the forecast anomaly matrix and increases the computational costs. Based on one of my previous remarks, I wonder if it would not be better to apply a standard EnKF or an ensemble-based smoother?

- p.4, l.130-131 "To generate the ensemble, we use snapshots from a multi-year model simulation without DA to create a set of n_{pc} leading principal component": Even if it is problem-dependant, I think that the authors should provide information on the length of the time window used to pick up the snapshots.
- p.5, l.143-144 "we use $n_{pn} = 25$": Could the authors mention the percentage of variance it was kept with 25 principal components? It would help the reader to better interpret this value.
- p.5, l.146-149 "In order to avoid negative values in any variables representing concentrations, we further enforce a minimum threshold of 0.001 $[\cdots]$ value.": I wonder why the authors did not use a log-transformation as they did with the 4dVAR algorithm they used in the numerical experiments. Could the authors explain this choice?
- p.5, l.150-152 "An ensemble regeneration can be benificial if the first ensemble member, the only ensemble member updated by observations, is steered away from the remaining ensemble". I wonder which metric the authors used to detect such phenomenon (variance, quantiles?). I think that this information should be present in the manuscript.
- p.5, 167-168 "the matrix that is inverted in Eq. (1)": In practice, I presume that the matrix is not inverted. Eq. (1) refers to the resolution of a single linear system. The sentance should me modified.
- p.6, l.193-200 "In addition to the spatial localization described above, we also consider the DA impact resulting from variable localization, $[\cdots]$ based on improper correlations in the ensemble": First I think that the authors must add a reference to Kang et al (2011), where variable localisation strategies were introduced in an atmospheric carbone cycle model. While such approaches may be usefull in practice for coupled systems in high dimension, for instance we may not want large corrections in the physical fields due to large misfits to observations in the biogeochemical model, I do not understand the rational to apply this approach between variables for the same component of the coupled system. This approach gives me the impression to going back in time when only the observed variables were corrected during the analysis, the unobserved ones being adjusted by the model dynamics during the forecast step. That's not what we expect from a multivariate data assimilation system.

  Kang et al : "Variable localization" in an ensemble Kalman filter: Application to the carbon cycle data assimilation, JGR Atmosphere, 2011.
- p.6, l.202-203 "Those correlation values associated with the different variables are multiplied by a factor of $l_{var} \in [0, 1]$": Furthermore, this strategy involves hyperparameters (one per combination of two variables) that need to be properly tuned. So, I wonder how can they be estimated? It is a serious issue..
- p.6-7 2.5 Splitting the computation: I think that this subsection must be removed from the manuscript or added as an appendix. I am not sure that the approach suggested is new regarding the complexity of operation DA system involving a lot of HPC strategies to design and run the system on supercomputers.
- p.7, l.254-255 "we have a test bed to assess the 4dEnOI implementation in comparison to a 4dVar benchmark implementation": The motivation for comparing the performances of the 4dEnOI to the 4dVar are not clear to me. I would have

expected ensemble-based methods like a static 4dEnOI, a EnKF and an ensemble-based smoother. Why a 4dVar that may not use an ensemble? I think that the authors should better motivate this choise.

- p.7, l.260-263 "Although, in the following, we assess [$\cdots$] largest difference in configuration between the DA two systems": It is not clear from the manuscript what is implemented in the 4dVar, but a second main difference between the two systems may be the modelling of the **B** matrix: one uses an ensemble for the 4dEnOI and the other a static matrix (I presume). If think that more information on the modelling of **B** in the 4dVar system are required.
- p.8, l.282-283 "we focus on the reduction in a cost function based on a weighted model-observation misfit ": I am sorry, but this diagnostic does not allow to conclude about the performances of the DA systems. There are too many hyper-parameters in both DA systems. So it is clear that a configuration of them will results in a decrease of the model-observation misfit. The evolution of the errors for the unobserved variables is crucial, notably with biogeochemical models. It is not rare to observe an improvement of the surface phytoplankton concentration while the errors in the nutrient concetrations are increasing over time (with seasonal cycle). The authors must assessed the performances of the systems with observations that have not been assimilated. Some in situ data must be available in this area and must be used for the intercomparison.
- p.9, l309-312 "while the 4dVar DA system creates [$\cdots$] especially chlorophyll-a": The impact of the modelling of **B** should be added in the discussion regarding the 4dVar system. We note on figure 4 that the NO_3 component of the increment is really low, implying no correction on this variable. The authors provide the information latter in the text.
- p.9, l.320 "the 4dVar increments created via tangent linear and adjoint dynamics": Again, the modelling of **B** can have a strong impact on the increments. It should be mentionned here as well.
- p.9 l.336 - p.10 l.339 "the lower prior error values 4dEnOI [$\cdots$] beneficial dynamical feedback into the next DA cycles": That's not true. Because the diagnostics are computed from the observed variables only, we can not guess what happens for the unobserved, especially the nutrient. Depending of the configuration of the DA systems, it is possible to control the dynamics of the errors in the observed variables, while having a damage of the unobserved variables. That's why the authors must compute diagnostics with additional data.
- p.10, l.362-363 "In our experiments, we use relatively strong variable localization with $l_{var} = 0.1$ when variable localization is applied": So basically, the best set up of the experiments is obtained when the cross-corellation among biogeochemical variables are removed. Waht the point to apply multivariate analysis schemes if ones needs to apply trick to make them monovariate? What does highlight these values is that the covariance matrix estimated from the ensemble is not relevant. Rather than trying to stick to the observations, I would rather try to improve the strategy to estimate the forecast error covariance matrix in an EnOI framework, or migrate to nonlinear updates.
- p.10, l.365-366 "With variable localization applied to all variables in L1, the prior and posterior errors increase by a few percent from the 4dEnOI reference": Could the authors explain why the performances of the DA system are degraded in the physical components with this variable localization strategy? To my point, adding hyperparameters that are tuned offline without a clear statistical rational, to compensate for sampling errors in the forecast error covariance matrix or nonlinera correlations among variables, does not seem to be relevant, neither in an EnKF nor an EnOI framework. I wonder what are the performances of a system with $l_{var} = 0$ (no cross-corellation among biogeochemical variables)? Would it be as good as the reference?

- p.11, l.399-400 "by reducing the error in the matrix inversion": In an EnOI framweork, there is no reasons to inverse a matrix, because ones needs to solve only one linear system. The authors may consider to reword the sentence.
- p.12, l.419-426 "In most cycles, the second increment further derceases the cost function, but in some cycles the cost function increases [$\cdots$] in the model-observation difference $\mathbf{y} - \mathbf{Hx}$." Could it not be related to the fact that the Gauss-Newon algorithm has not guarantee of global convergence regarding the minimization of the cost function?
- p.12, l.446 "or the inverse matrix": Again, it is must cheaper to perform a factorization of the matrix, and solve few systems than computing the inverse of the matrix. Please reword.
- p.12-13 "3.5 Ensemble regeneration": The authors apply a strategy for which a generation of the ensemble is applied for predefined cycles (2 and 3). I wonder why they did not try to detect when it was required (which statistics to detect it?) rather defining a priori cycles. We might expect to note divergence between the ensemble and the updated member after several cycles as well. I think the authors should better motivate this choice.
- p.14, l.547-548 "a positive effect that is difficult to measure due to the lack of observations for these variables": I think that's the main issue. Because this variable inflation, and the way to tune the hyperparemeters, which have no mathematical grounds, it is really unsatisfactory to not see diagnostics on unassimilated variables. That's why I urge the authors to provide diagnostics with independant data.
- p.14, l.554-560 "Similarly, we tested a few variables localization configurations [$\cdots$] compared to nutrient-zooplankton covariances": This affirmation is somehow speculative and unfortunately the authors do not provide strategies to compute the localization coefficients. It seems to improve the results for this specific configuration, based on statistics built on the assimilated observations. So it is not clear if the unassimilated variables are improved, and at which extend this approach can be generalized.
- p.15, l.576-577 "We note that an EnKF update in our implementation would still be computationally more costly [$\cdots$] rather than a single model state": This is true, but considering that the forecast step is the most expensive, the regeneration of the ensemble requires a SVD (or a subspace method for eigenvalue), and finally that the EnOI approach does not update the ensemble anomaly, I wonder at which point an EnKF with the same ensemble size remains a good alternative. Unfortunately, there is no comparisons to an EnKF in the numerical experiments.
- p.15, l. 581-583 "because it requires the inversion of a $n_{obs} \times n_{obs}$" matrix. Numerically, more efficient techniques to compute or approximate this matrix inversion, werre not investigated in this study": Because there is only one system to solve in the EnOI framework, the cheapest and most robust strategy to use is simply to solve the system: Cholesky factorization, preconditioned conjugate gradients.
