# Supplementary material for: Ensemble optimal interpolation for adjoint-free biogeochemical data assimilation
Source: PLoS One. 2023 Sep 5;18(9):e0291039. doi: 10.1371/journal.pone.0291039 (PMC10479889; doi:10.1371/journal.pone.0291039)
Supplement: Attachment — Submitted filename: response_letter.pdf [file pone.0291039.s003.pdf]

Note: In their comments, all reviewers make some general points that we aim to address with this note. The reviewers mention other DA techniques, like the EnKF, and ask for a comparison between EnKF and the 4dEnOI implementation. We agree with the reviewers, that we did not emphasize the benefits of our 4dEnOI implementation compared to the EnKF, omitting some important information. Our manuscript was focused on the comparison of the 4DEnOI technique to 4dVar, and the benefits of not having to create tangent linear and adjoint code. In particular, we did not pay enough attention to the "static ensemble" aspect of the 4dEnOI technique. Indeed, the techniques as it was presented in the initial submission of this manuscript can use a static ensemble for all experiments except the "ensemble regeneration" presented in Section 3.6. That is, if model solutions for ensemble members 2-25 are stored (model state at the start of every DA cycle, and model solutions at the observation locations), only the first ensemble member needs to run for a new experiment. Thus the 4dEnOI technique can achieve the results we present at a fraction of the computational cost of running a full ensemble. Based on the comments of the reviewers, we have included this information in the manuscript, which better motivates why we are using the 4dEnOI technique instead of the EnKF in this application, addressing one of the the main criticisms of the reviewers. Though a carefully configured EnKF implementation may yield better results than the 4dEnOI technique we present, this outcome would require considerably higher computational effort. We further think that the comparison of its results to the "benchmark" 4dVar implementation is enough comparison to quantify the results of the 4dEnOI implementation. 4dVar is a mature, advanced method for data assimilation and comparisons between 4dVar and EnKF have been made by other research groups. The main reason why we use 4dVar is that we have an existing 4dVar configuration for this model, and a comparison with an EnKF would require a new implementation and tuning of the technique.

Comments of the editor and responses

comment: I overall agree with the reviewers comments that the proposed methods not only lack mathematical rigorous but their rationales are not explained nor well justified.

We thank the editor for the comments. In response to this comment and that of the reviewers, we have reframed the introduction and description of the 4dEnOI to better justify and describe it. The use of a passive ensemble, which was left out of the description of the methods before our revisions, better justifies the use of 4dEnOI over an EnKF technique. Using the first, and only non-static, ensemble member in the computation of ensemble statistics is better motivated in the new Section 3.3. We have summarized the main changes to the manuscript in the note above.

... As also suggested by the third reviewers, the analysis of the results is also not complete.

In response to this comment and those of reviewer 3, we expanded the analysis of the results, examining spurious correlations between variables (see updated Fig 6 and accompanying text), thus better justifying the use of variable localization in this application. We further added subsection Section 3.3 to the results, motivating the inclusion of the first ensemble member in the ensemble statistics. See the responses below for a detailed description of the changes to the manuscript based on the comments of reviewer 3.

Comments of reviewer 1 and responses

major comments

comment: This manuscript proposed a four-dimensional ensemble optimal interpolation (4dEnOI) for a simple marine ecosystem model. The 4dEnOI uses regenerated ensemble ICs to launch ensemble forecasts and incorporate flow-dependent background error covariances. Various localizations, including variable localization, horizontal and vertical localization, are examined. Results show that 4dEnOI performs similarly to 4DVAR. The 4dEnOI highly depends on covariance localization, and it can be improved with multiple iterations. The manuscript is well written, but there are places where clarifications and explanations are needed. Please see my comments below.

We thank the reviewer for the thoughtful comments, please see our response below.

(M1.1) comment: It is unclear how the algorithm proposed here is a four-dimensional one. It mentioned that "observations that vary both in space and time (and are thus 4-dimensional)". But if the observations within an assimilation window is assumed to happen at the assimilation time, or a FGAT is used, it is not really a four-dimensional method. Are multiple time slices of state variables used in an assimilation window?

In response to this comment and the comments of other reviewers, we have expanded the description of the 4DEnOI implementation. The technique is four-dimensional, in that it computes ensemble statistics from the model solutions at the observation locations (in time and space) within a given assimilation window. This approach is easy to implement and works with a static ensemble, as long as the ensemble solutions at the observation locations $H\mathbf{X}$ are stored alongside the initial conditions (model state at the start of every assimilation cycle). We have now clarified this in the manuscript:

revised text (Section 2.1, par. 4): The ensemble, which is providing estimates of the true model covariances, is used to update the first ensemble member with observations based on Eq. (1). Unlike many other ensemble-based DA techniques like the EnKF, the remaining ensemble is not updated with data directly, and can remain static. When using a static ensemble, the initial conditions (full model state at the start of each DA cycle) and the model solutions at the observation locations need to be stored for each ensemble member.

(M1.2) comment: The authors state that "What sets our implementation further apart from other 4dEnOI approaches is that it does not use a static ensemble of model simulations. Instead, it is based on flow-dependent statistics which are obtained from a dynamical ensemble using spatial and variable localization to obtain more reliable statistics." As described in section 2.2, for each assimilation cycle, the initial ensemble ICs are generated by projecting the updated x1 on climatological PCAs with perturbations. Then the ensemble ICs are integrated forward. The climatological PCAs approximates the leading components of a static background error covariance, thus the forecasted ensembles approximate the static background error covariance, rather than the common EnKF's flow-dependent background error covariance. Moreover, ensemble model integration requires much more computational cost than the assimilation. Since an ensemble model integration is used, why not just use the common EnKF?

We agree with the reviewer that using an EnKF could likely improve the results from the 4dEnOI technique presented here. As stated in the note at the top of this document, the technique as it was presented in the initial submission of this manuscript can use a static ensemble for all experiments except the "ensemble regeneration". We have changed the wording throughout the manuscript to make this aspect clear. Thus, the 4dEnOI technique only requires a single "extra" model evaluation, instead of running a full ensemble.

... One more comment is whether ensemble mean equals x1? If not, the ensemble perturbations are biased.

Because, $\mathbf{x}_1$ is the only non-static ensemble member, we use it to represent the state estimate, the remaining ensemble is not adjusted. We hope that this point is more clear in the updated manuscript.

(M1.3) comment: Are the horizontal localization of 1 degree and vertical localization of 300 m optimal? Since an exponential localization function (Eq. 8) is used, the observation impact does not go to the exact 0 at a certain distance?

The selected values are not strictly optimal, but very likely within half an order of magnitude of an optimal value. In order to keep things reasonably simple, we used a "half an order of magnitude approach" to select the values. This information is now included in the manuscript:

revised text (Section 2.3, par. 3): In order to select suitable length scale values, we employed a "half an order of magnitude approach": first, using no vertical localization, we tested horizontal length scales of 0.1°, 0.3°, 1°, and 3°, increaseing by half an order of magnitude. We then selected the one that minimized the prior error in 8 DA cycles (run with a static ensemble in the same setup presented in Section 3.1). If the lowest or highest value had the lowest error, the series would have been extended. In the second step, using the 1° horizontal length scale, we selected a vertical length scale using the same procedure, testing values of 10 m, 30 m, 100 m, 300 m, and 1 000 m.

Regarding the implementation of the spatial localization, the weights do not go to the exact zero at a certain distance. A threshold like this could easily be implemented, but we have no reason to presume that it would result in a significant reduction in the computational cost or improvement in results in our implementation.

(M1.4) comment: Section 2.5, is the parallelization described here similar to Anderson and Collins 2007 (Scalable implementations of ensemble filter algorithms for data assimilation)?

The approach appears to be similar, but far less sophisticated than the approach presented in Anderson and Collins (2007), where not only the state (as in this manuscript), but also the observations are being divided and processed separately. Based on the comments from other reviewers, we have moved the section in question to the Supporting Information document and mention (Anderson and Collins 2007).

(M1.5) comment: Figure 3b, it is interesting that 4dEnOI has smaller errors than 4DVAR. 4DVAR has temporal propagation of background error covariance matrix and localization matrix during an assimilation window, which should be beneficial. Please explain why 4dEnOI has advantages over 4DVAR here?

It is difficult to know the exact reasons for this, it has likely to do with the modeling of $\mathbf{B}$. The diagonal entries of $\mathbf{B}$ in the 4dVar implementation are based on variability in a long simulation, off-diagonal elements are the set using length scales. We reduced the diagonal values for *unobserved* variables (see Mattern et al. 2017). Given that the 4dVar technique works better for physical than for biogeochemical variables (see colored bars in Fig 3), it is likely that the 4dVar technique is hampered by non-optimal increments to unobserved variables, partly due to non-optimal entries of $\mathbf{B}$ for these variables. There is no easy solution to this problem, and unobserved variables are currently an unavoidable problem in biogeochemical modeling and DA.

(M1.6) comment: l365-374, with or without variable localization, there are no significantly different performances for 4dEnOI. Intuitively, variable localization could exacerbate the imbalance of analysis increment and subsequent model forecast. To quantify the imbalance, can the surface pressure be verified?

Variable localization was not applied to physical-physical correlations, hence it did not substantially modify the result of the DA update for the physical variables. This circumstance was not entirely clear in the manuscript

and in response to this comment, we have added this information in the description of the method.

(M1.7) comment: Section 3.4, with multiple iterations, how's the computational cost increase for 4dEnOI?

Each additional iteration has the same computational cost as the first (with the exception that only the first ensemble member needs to be run again, even in case of an ensemble regeneration). We have added this information to the manuscript:

revised text (Section 3.5, par. 2): The computational cost for each new iteration (consisting of a model simulation and an 4dEnOI update), is the same as that of the first iteration when a static ensemble is used.

specific comments

(S1.1) comment: l9, please add some references for 'variational DA techniques'.

We agree, and in response, have added relevant references that use coupled physical-biogeochemical models.

(S1.2) comment: l13, please add some references for 'ensemble-based DA'.

We added references here as well, moving them up from the following paragraph.

(S1.3) comment: l95, X should have dimension of 'n_state * n_ens'.

Good catch, we corrected this mistake.

(S1.4) comment: l98, HX should have dimension of 'n_obs * n_ens'.

Corrected.

Comments of reviewer 2 and responses

comment: The authors present a biogeochemical ocean data assimilation (DA) study in the California Current System. The DA framework uses a variant of the ensemble optimal interpolation (EnOI) scheme with several modifications to the original EnOI algorithm. In particular, the static background covariance is not static anymore, a variable localization is utilized and furthermore a scaling factor is used to limit the impact during the update. The authors also propose a way to regenerate the ensemble in case the first member starts drifting away from the rest of the ensemble. I find the new method quite ad-hoc with very little mathematical rigor and limited scientific impact. The authors however do compare its performance to an already existing 4DVAR solution. The results show that the proposed 4dEnOI system can produce comparable performance to 4DVAR. I recommend rejection of the article.

We thank the reviewer for the helpful comments. Please see the note at the top of this document regarding the use of a static ensemble.

(M2.1) comment: I am struggling to find a motivation for the new EnOI method. We know for a fact that an EnKF with a full dynamic covariance outperforms the EnOI with a static (and sometimes seasonal) covariance. The whole motivation for using EnOI is to cut down on the massive computational burden that one pays by running a full ensemble. The authors are in fact integrating the entire ensemble using the model so why not simply use the EnKF? We know that it gives better estimates than a single-member EnOI. I find this quite troubling. I understand that the simple NPZD-type system is quite cheap to integrate forward in time, but this is rarely the case in large and more realistic models.

We agree with the reviewer's point, which was also commented on by the other reviewers. As explained in the note at the top of this document in more detail, we now better motivate the 4dEnOI technique, which can be run using a static ensemble and thus at lower computational expense than an EnKF. Another reason to use the 4dEnOI technique is that it is less complex than an EnKF, despite still containing lots of adjustable parameters, as pointed out in Comment M2.3.

(M2.2) comment: My second major take on the method is the issue of only updating the first member. I find it difficult to justify this choice. If the entire ensemble is dynamic, why not update all the members. I can see scenarios in large models where only updating the first member can cause inconsistencies leading to divergence and model instabilities. The authors further suggest an ensemble regeneration technique where the new members are sampled around the posterior (i.e., the first member). This feels counter-productive adding an extra layer of complexity to an already complex framework. On the other hand, the EnKF gives you an ensemble after each assimilation cycle that is centered around the posterior estimate which happens to be the mean.

This comment is addressed by the note at the top of the document. The ensemble is static, except in the ensemble regeneration experiments, where a dynamical ensemble leads to slightly improved results. Again, we thank the reviewer for the comment and regret that the lack of information about the ensemble caused confusion.

(M2.3) comment: It seems to me that a lot of tuning is required for the method to become competitive. Tuning includes optimizing the scaling factor alpha (also known as damping factor in the literature e.g., Hendricks Franssen and Kinzelbach 2008), finding the right variable localization and deciding when to regenerate the ensemble. This makes the technique less desirable in other applications.

While it is true that there are lots of tunable parameters – the localizations scales centrally among them – we do not believe that there is a DA technique, suitable for high-dimensional biogeochemical models, that has fewer parameters. The 4dVar technique we employ uses a similar set of length scales to specify background covariance values, while an EnKF implementation typically requires a setup similar to the 4dEnOI with the potential additional implementation of covariance inflation.

(M2.4) comment: As far as variable localization goes, what is a good strategy to select lvar? Ideally, using a statistically sound ensemble lvar should be set to 1 and it is left to the model correlations to determine the impact of the update on the variables in the state. I'm just thinking in a large state where you have tens of variables tuning this variable localization would be a hassle.

We agree with the reviewer that finding a suitable value for l_{var} can be difficult. We used a similar approach to that employed to find values for the spatial localization length scales: starting with $l_{\mathrm{var}} = 1$, we successively reduced the value by half an order of magnitude. In response to this comment, we have added this information to the manuscript:

revised text (Section 2.4, par. 2): To select values for l_{var}, we employ a similar approach as for the spatial localization and starting with a value $l_{\mathrm{var}} = 1$, successively decreased it by half an order of magnitude, testing 0.3, 0.1, and 0.03, and selected the value resulting in the loweset prior error in 8 DA cycles. We further distinguish between physical-physical variable correlations (such as temperature-salinity correlations) and others (physical-biogeochemical and biogeochemical-biogeochemical correlations, such as temperature-NO_3 and NO_3-phytoplankton correlations, respectively). A finer-grained approach of weighting the correlations between variables based on their "dynamical distance" – for example, NO_3 and phytoplankton are more closely linked than NO_3 and zooplankton – is not tested in this study.

(M2.5) comment: I suggest rewriting section 2. Many points need to be summarized and perhaps removed and citations to relevant literature could be added instead. For instance, ensemble generation using PCA by projecting onto the leading modes is well-known and has been introduced and discussed extensively in the literature. The first paragraph in Section 2.3 about localization can also be omitted. After more than 20 years, DA readers should be quite familiar with localization and the need for it. Section 2.5 describes a splitting strategy to make the Kalman update more efficient. Operational centers and many DA software have been using different ways to make the update more efficient. Splitting is something that has been used in many other systems. For instance, the TOPAZ system (Sakov et al., 2012) splits/slices the update by vertical layers.

We mostly agree with this comment and, in response, have attempted to shorten Section 3. Based on Comment S3.14, we have moved the computational aspects from the former Section 2.5 to a supporting document/appendix, where we now cite Sakov et al. (2012). While we added some information in response to the comments of the other reviewers, Section 2.2 and 2.3 are not long, and we believe it is beneficial for the reader not to have to consult other literature sources for implementation aspects that can be described in a few sentences. In response, we have added references to relevant literature to the PCA (Barth et al. 2008, Pan et al. 2014, Vervatis et al. 2016).

specific comments

(S2.1) comment: Abstract: Computationally suitable instead of suitable computationally.

We corrected this mistake.

(S2.2) comment: Abstract: its results instead it results?

Corrected.

(S2.3) comment: Line 55: and *is able to* accommodate changes.

Corrected.

(S2.4) comment: Line 68: variable localization is vague here. It could be described briefly here.

We have added more information and a reference:

revised text (Section 1, par. 4): Here, spatial localization refers to the practice of dampening ensemble-based estimates of correlations if they are spatially distant, which is commonly employed in ensemble-based data assimilation applications to correct the effects of small ensemble sizes (Houtekamer and Mitchell 2001, Anderson 2007, Hu et al. 2012). Variable localization performs a similar function, but reduces the correlations between "dynamically distant" model variables (Kang et al. 2011).

(S2.5) comment: Line 98: Shouldn't HX be Nobs x Nens?

Good catch; corrected.

(S2.6) comment: Line 105: Add a reference to motivate the use of alpha.

A good idea, we added a reference to (Hendricks Franssen and Kinzelbach 2008) using an EnKF and two EnOI applications (Counillon and Bertino 2009, Blyverket et al. 2019).

(S2.7) comment: Equations 3 and 4: I don't see the point of this approximation. Should these statistically be equivalent even though B is time-invariant? Adding time subscripts in your equations may help.

Here we mainly wanted to emphasize the rough equivalence of the terms. Because this may get more complex when considering different time indices, we decided the equation/approximation was more confusing than helpful, and removed it from the manuscript.

(S2.8) comment: Line 308: reveal instead of reveals.

Corrected.

(S2.9) comment: Section 3.4: I don't quite understand the iterative procedure. Again, adding equations here can be very helpful. I though you're iterating over the update equation at a single time. If you have to rerun the model starting from the posterior, doesn't that take you to the next cycle?

In response, we have tried to improve the description of the iteration, making more use of mathematical symbols (the equation governing the repeated update remains the same).

revised text (Section 3.5, par. 2): Each following iteration updates the previously updated state estimate. This step requires re-running the model with the previously updated state $\mathbf{x}_1^*$, in order to obtain $H\mathbf{x}_1^*$ which is then used to update the state again using Eq. (1). Each iteration thus uses a different initial state estimate and slightly modified ensemble statistics, because the first ensemble member has changed.

(S2.10) comment: Line 493: 4dEnOI or 4DEnOI. It's good to stick to one notation.

Good catch, we are now consistently using "4dEnOI".

Comments of reviewer 3 and responses

comment: In this paper, the authors present a variant of 4dEnOI with application to a coupled ocean-biogeochemical model. Compared to traditionnal EnOI, they suggest to run an ensemble in order to provide flow-dependent statistics. The main differences with ensemble-based Kalman filter and smoother is that only one member is updated, while the remain part of the ensemble evolves freely accordingly to the dynamics of each member. A strategy of regeneration of the ensemble is suggested when the observation-aware member tends to be steered awy from the ensemble. In this case, it imples the computation of the most dominant principal components of the forecast anomaly matrix to resample the distribution. Furthermore, the authors suggest a strategy of variable localization, introducing unknown hyperparameters that aim to weaken cross-corellation among variables. Numerical experiments are done with a coupled model encompassing the domain of the California Current System developped at UCSC. The biogeochemical model is a simple NPZD model coupled with ROMS and real observations are assimilated (satellite and in situ data). Finally, the authors compared the performances of several variants of their 4dEnOI with a 4dVar using a log-transformation.

The manuscript is well written and the topics is relevant, beacuse building data assimilation (DA) systems for coupled ocean-biogeochemical models remains challenging.

We thank the reviewer for the plentiful comments. Please see the note at the top of this document regarding the use of a static ensemble.

(M3.1) comment: However, this work has some weaknesses:

The diagnostics shown in the numerical experiments are computed with the assimilated observations. Unfortunately, we can not conclude on the benefits of the DA system only from what ones observes, because improving the misfit to assimilated observations does not imply an improvement of the unobserved variables. This is particlarly true in the framework of ocean biogeochemistry (impact on nutrient concentrations?). I think that the authors have to compare to ”independant” observations. Why not using the in situ data for the diagnostics, and so not assimilaing them? Evolution of the nutrient concentrations could also be shown. I am sorry, but just comparing the decreases in the observation term of the 4dVar cost function is not enough.

We agree with the general point of the reviewer that “independent” observations are very valuable, especially for biogeochemical variables for which no data is assimilated, such as the NO_3 variable in our model. Unfortunately, for those biogeochemical variables, there is very little or no data available. Even if there is some, it tends to be very sparse or cover only a small, not necessarily representative, part of the model domain. We have examined the use of climatological nutrient data in evaluating model output in Mattern and Edwards (2017), which can be useful to detect very unrealistic changes to the model state. In summary, we agree that it would make for an interesting study to more carefully evaluate the impact on “partially observed” variables – perhaps in combination with a more sophisticated approach to perform variable localization based on “dynamical distance” – but we think such an approach is not necessary to demonstrate the utility of our approach and is beyond the scope of this manuscript. Here, we lay the groundwork, introduce the technique, assess the value of horizontal, vertical (which is not used in many studies), and variable localization (used in very few studies), examine the benefits of including a dynamical member in an otherwise static ensemble (see new Section 3.3), and compare it to an established DA system. After this, we plan to use 4dEnOI as a test bed for further, more involved studies.

It is important to emphasize that biogeochemical models consist of linked state vector elements through which mass cycles. For example, inorganic nitrogen is incorporated into phytoplankton through growth, then transferred to zooplankton through grazing, and ultimately returned to inorganic nutrients via particulate and dissolved organic matter pools. As a result, observations of phytoplankton, (i.e. surface chlorophyll) can meaningfully constrain other variables through adjustment of total nitrogen available in the system and through forward model dynamics in subsequent cycles. While our diagnostics are indeed computed for assimilated observations, we focus primarily on the *prior* error (i.e., a diagnostic based on the assimilated observations, but *before* they are assimilated). Thus, these observations are independent in that they are used for evaluation

before being assimilated. In addition, they often occur in locations where observations in previous assimilation cycles did not exist, due to variable satellite trajectories and cloud cover. Furthermore, the prior estimate of phytoplankton concentrations (and thus surface chlorophyll) is influenced by other adjusted biogeochemical variables, and does not imply persistence. This approach is not as good as the use of observations from other instruments, but offers considerable insight, and is preferable to the use of the posterior error, which we also report.

(M3.2) comment: The 4dEnOI with variable localization, as suggested by the authors, is based in questionnable choices. The first one is to run an ensemble in the forecast step, but updating only one member. In a lot of operational ensemble-based DA systems, the most expensive task is to run the ensemble during the forecast step, not to apply the analysis step. Considering that a regeneration of the ensemble may be required, I am not sure that the benefits of the 4dEnOI are that large. Unfortunately, there is no comparisons to an EnKF nor an EnOI in the numerical experiments.

As stated in the note at the top of this document, we modified the manuscript based on this and other comments to clarify and better motivate our 4dEnOI implementation. We apologize that this aspect was not emphasized in the first version, but all the main experiments, except for the ensemble regeneration (Section 3.6) were indeed using a static ensemble, except for the dynamically adjusted first ensemble member. In response, we have further added a section in which the first ensemble member in not included in generating ensemble statistics (Section 3.3), akin to a typical static ensemble 4dEnOI implementation.

... The introduction of the variable localization is very questionnable, and seems to be introduced to mitigate errors in the estimation of the forecast error covariance matrix. Because it introduces unknown hyperparameters (no strategies are introduced to estimate them), and good performances are obtain with low correlation among the biogeochemical variables (monovariate analysis scheme?), I really need a better intercomparison among the state-of-the-art algorithms to start to be convinced.

First off, we now state in more places that variable localization is not applied to physical-physical correlations, but only to physical-biogeochemical and biogeochemical-biogeochemical correlations. We show the results with and without variable localization, and also the (negative) effect of applying variable localization to all variables and not distinguishing between physical-physical or physical-biogeochemical covariances. In response to this comment, we have added a description of how we arrived at a value of $l_{\text{var}} = 0.1$ to Section 2.4. Additionally, we also examine the correlations between model solution at the observation locations and all (including unobserved) variables with and without variable localization (insets in Fig 6e,f). The results indicate that the variable localization, very much like spatial localization, eliminates the effects of some unrealistically high correlations, but that a more advanced approach for variable localization could yield better results. This point has now been emphasized in the discussion:

revised text (Section 4, par. 5): Similarly, we tested a few variable localization strengths before our experiments, but did not examine the effect of a more fine-grained approach, applying different variable localization strengths to different variable combinations. Similarly, we tested a few variable localization strengths before our experiments, but did not examine the effect of a more fine-grained approach, applying different variable localization strengths to different variable combinations. For example, a typical NPZD biogeochemical model simulates a nutrient → phytoplankton → zooplankton → detritus → nutrient cycle, and the results in Section 3.4 indicate that there may be benefit in applying weaker variable localization to more closely related variables, such as nutrient-phytoplankton covariance terms, compared to nutrient-zooplankton covariances.

As stated in response to Comment M3.1, we may consider a more sophisticated approaches and analyses of variable localization in future studies.

(M3.3) comment: I am surprised that no comparisons are made against ensemble-based method like an EnKF, an ensemble smoother, a hybrid EnOI or a static 4dEnOI. Regarding the EnKF and the hybrid EnOI, they can assimilate asynchronous observations. So I wonder why the authors did chose a 4dVar with a possible static **B**. The authors should explain why they did not choose at least one of these method to highlight the benefits of their approach.

We agree that it is useful to compare new DA techniques to more established ones. With the addition of Section 3.3, we have now included the comparison to a more typical 4dEnOI implementation with a fully static ensemble. Why did we not include other techniques? Simply because we do not have them readily available. We have been working with the 4dVar for years, have experience with and have applied this technique to several biogeochemical models. Implementing a full EnKF or similar techniques from scratch would require calibrating it for this application, just as the 4dEnOI, but likely with even more hyperparameters. In addition, other authors have made comparisons between EnKF and 4dVar approaches (see, e.g., Moore et al. 2020), and we believe that evaluation against a mature 4dVar system is satisfactory.

specific comments

(S3.1) comment: Abstract: "our 4dEnOI reference implementation performs similarly well to the 4dVar benchmark in lowering the observation misfit": I wonder why the authors do not compare the performances of their approach with data that have not been assimilated. Improving the misfit to the observations that are assimilated is the minimum that ones expects with a data assimilation system.

As we explain in response to Comment M3.1, few "independent" observations exist, and most biogeochemical variables are currently unobserved or too sparsely observed to be of use for model-data comparison.

We further think that the prior error, which is based on the observations before they are assimilated, and that we use as a main diagnostic, can be very useful in assessing skill in DA systems. Large discrepancies between forecast and posterior (analysis) error may indicate issues like overfitting or deleterious increments to unobserved variables, which slowly feeds back to an observed one.

(S3.2) comment: p.1, l.4-6 "DA is applied to coupled physical-biogeochemical ocean models, where it yields large improvments in the model's state estimates (Edward et al. 2015, Mattern et al. 2015)": The authors refer only to their previous works while this topic has been investigated by many other groups. I would suggest to add references.

We agree, and have added new references.

(S3.3) comment: p.1, l.9-15 "Variational DA techniques, one group of widely-used DA techniques [...] Ensemble-based DA techniques, the second group of widely used DA techniques, rely on statistics [...] estimates": This splitting into two groups is not realy relevant now, because of the development en ensemble-based varational approaches (hybrid B matrix, EnVAr, Iterative EnKS, 4DEnvar, etc..). I would rather reword with one group relying on optimal control theory (4DVar) and the second one from the linear estimation theory (Kalman filter).

While hybrid techniques are becoming indeed more popular, we think that for readers not very familiar with DA, the suggested description (based on optimal control theory vs. based on linear estimation theory) will not be very helpful. In response, we now mention that hybrid techniques exist and are becoming more prevalent:

revised text (Section 1, par. 1): Hybrid techniques, that combine elements of variational and ensemble-based DA, exist and are becoming more prevalent (e.g., Wang and Lei 2014, Yaremchuk et al. 2017). Based on the technique, they share advantages and disadvantages of the underlying methodologies they are based on.

(S3.4) comment: p.2, l.67 - p.3, l.74 ”Instead, it is based on flow-dependant statistics which are obtained from a dynamical ensemble [...] making it suitable for complex biogeochemical models with many variables and consequently a very high-dimensional state vector.” The authors suggest to dynamically run an ensemble to estimate the forecast mean and error covariance matrix within the DA process as done with an Ensemble Kalman smoother. However, they suggest to update only one member, and not the full ensemble. I am not sure to understand the rational of such approach. Usually, it is the forecast step that is the most expensive in terms of computational cost and time in an ensemble-based DA system for coupled models in high dimension. The analysis step remains cheaper compared to the forecast step. So having the ensemble, I do not think it changes that much the total computational cost and time to update one member rather than the full ensemble, and it would allow the estimation of the analysis error covariance matrix. Could the authors comment on my remark ?

The reviewer is correct, our description of the algorithm left out an important detail. The 4dEnOI algorithm, as we presented it, could indeed make use of a static ensemble, it can thus save considerable computational expense. The static nature of the remaining ensemble also explains the update of the first, and only non-static, ensemble member. We address this comment in more detail in the note at the top of the document.

(S3.5) comment: p.3, l.80 ”the EnOI implementation to a 4dVAR-based DA system”: The comparison to a 4dVar algorithm is interesting. However, this system does not use a flow-dependant error covariance matrix. I know that it is difficult to assess the performances of their 4dEnOI against all the existing algorithms, but could the authors better justify why they did not compare their results with a standard 4dEnOI or an ensemble-based Kalman smoother?

As we mention in response to Comment M3.3, we have been working with a 4dVar system for years and have used it with the same biogeochemical model. Thus, it seemed like a natural choice to use it as a benchmark, rather than a second system that we would have had to develop from scratch.

(S3.6) comment: p.4,l.119-126 ”Unlike many other ensemble-based DA techniques like the EnKF, the remaining ensemble is not updated with data directly, but run alongside the first ensemble member [...] passively updating the full ensemble with observations”: With the update of only one member, it is not posible to reduce the uncertainty in the solution through the estimation of the analysis error covariance matrix. Furthermore, it raises issues in the dynamics of the member wich is updated with the observations, and the other members of the ensemble. The authors suggested to regenerate the ensemble to remedy for these issues. However, it requires to compute the SVD of the forecast anomaly matrix and increases the computational costs. Based on one of my previous remarks, I wonder if it would not be better to apply a standard EnKF or an ensemble-based smoother?

This comment is similar to Comment S3.4. Based on these two and other comments, we have improved the description of the algorithm. Because a static ensemble is used (note at top of document), it is now clear why the full ensemble is not updated.

(S3.7) comment: p.4, l.130-131 ”To generate the ensemble, we use snapshots from a multi-year model simulation without DA to create a set of n_{pc} leading principal component”: Even if it is problem-dependant, I think that the authors should provide information on the length of the time window used to pick up the snapshots.

This simulation had a length of 5 years. In response, we have added this information to Section 2.2 and the caption of Fig 2.

(S3.8) comment: p.5, l.143-144 ”we use $n_{pn} = 25$”: Could the authors mention the percentage of variance it was kept with 25 principal components? It would help the reader to better interpret this value.

The PCs kept $> 91\%$ of the variance, and we have added this information to the manuscript.

(S3.9) comment: p.5, l.146-149 "In order to avoid negative values in any variables representing concentrations, we further enforce a minimum threshold of 0.001 [...] value.": I wonder why the authors did not use a log-transformation as they did with the 4dVAR algorithm they used in the numerical experiments. Could the authors explain this choice?

There are two reasons for this choice: (1) We wanted to keep our first implementation simple and examine the capabilities of the system without having to implement log-transformed variables. (2) The back-transformation using exp can amplify noise, which may require additional safeguards. Our priority was not to examine the advantages and disadvantages of log-transformed biogeochemical variables, in future implementations, we may examine this topic.

(S3.10) comment: p.5, l.150-152 "An ensemble regeneration can be beneficial if the first ensemble member, the only ensemble member updated by observations, is steered away from the remaining ensemble". I wonder which metric the authors used to detect such phenomenon (variance, quantiles?). I think that this information should be present in the manuscript.

Initially, this statement was based on intuition and the knowledge that updated ensemble member one which was previously close to the center of the ensemble (based on the way the ensemble is generated), has received DA updates, bringing it closer to the observations. As mentioned in the response to earlier comments, we have added Section 3.3 to the results, which performs DA using the same 4dEnOI procedure, but excluding the first, and only DA adjusted ensemble member from the calculation of the ensemble-based statistics. The resulting worsening of results indicates the value of ensemble members closer to the observations. In response to this comment, we have now added this point to the motivation of the ensemble regeneration experiments.

(S3.11) comment: p.5, 167-168 "the matrix that is inverted in Eq. (1)": In practice, I presume that the matrix is not inverted. Eq. (1) refers to the resolution of a single linear system. The sentance should me modified.

We thank the reviewer for this useful comment. A Cholesky decomposition of the matrix required the change of a single line of code and sped up that line by almost a factor of 3. In response to this comment, Comment S3.23, S3.25, and S3.30, we have modified the manuscript in places, mention the use of the Cholesky decomposition, and removed a mention of the matrix inversion as suggested in the comments.

(S3.12) comment: p.6, l.193-200 "In addition to the spatial localization described above, we also consider the DA impact resulting from variable localization, [...] based on improper correlations in the ensemble": First I think that the authors must add a reference to Kang et al (2011), where variable localisation strategies were introduced in an atmospheric carbone cycle model. While such approaches may be usefull in practice for coupled systems in high dimension, for instance we may not want large corrections in the physical fields due to large misfits to observations in the biogeochemical model, I do not understand the rational to apply this approach between variables for the same component of the coupled system. This approach gives me the impression to going back in time when only the observed variables were corrected during the analysis, the unobserved ones being adjusted by the model dynamics during the forecast step. That's not what we expect from a multivariate data assimilation system. Kang et al : "Variable localization" in an ensemble Kalman filter: Application to the carbon cycle data assimilation, JGR Atmosphere, 2011.

We thank the reviewer for making us aware of this publication and have added a reference to the Introduction, where variable localization is first mentioned.

Concerning the reservation towards employing variable localization, we share the concern of the reviewer that the update to unobserved variables may not be optimal. Yet, in biogeochemical DA most variables are unobserved in typical application and the advances in biogeochemical observing systems is easily outweighed by

the increase in the number of variables in modern biogeochemical models, so this will be an issue going forward.

At the same time, we now readily accept spatial localization in DA systems to remove the effect of spurious correlations in small ensembles. When spurious correlations also affect the correlations between different variables in small ensembles, one can easily argue for the use of variable localization. Perhaps a more sophisticated approach than using two values ($l_{\mathrm{var}} = 1$ or 0.1) could be employed, but as stated before, this is for a future study to investigate.

In response to this comment and Comment M3.2, we have added inset panels to Fig 6e and f, showing the high correlations between some not strongly dynamically linked variables. As stated above, the results may motivate a more elaborate approach for variable localization, which we may investigate in a separate study.

(S3.13) comment: p.6, l.202-203 "Those correlation values associated with the different variables are multiplied by a factor of $l_{var} \in [0, 1]$": Furthermore, this strategy involves hyperparameters (one per combination of two variables) that need to be properly tuned. So, I wonder how can they be estimated? It is a serious issue..

We share the view of the reviewer, our aim was to keep the implementation simple. In response to this comment and Comment M2.4, we have added the description of our approach for selecting the values of l_{var} and that of the localization length scales:

revised text (Section 2.4, par. 2): To select values for l_{var}, we employ a similar approach as for the spatial localization and starting with a value $l_{\mathrm{var}} = 1$, successively decreased it by half an order of magnitude, testing 0.3, 0.1, and 0.03, and selected the value resulting in the lowest prior error in 8 DA cycles.

(S3.14) comment: p.6-7 2.5 Splitting the computation: I think that this subsection must be removed from the manuscript or added as an appendix. I am not sure that the approach suggested is new regarding the complexity of operation DA system involving a lot of HPC strategies to design and run the system on supercomputers.

We followed the suggestion of the reviewer and moved this section to an SI document/appendix.

(S3.15) comment: p.7, l.254-255 "we have a test bed to assess the 4dEnOI implementation in comparison to a 4dVar benchmark implementation": The motivation for comparing the performances of the 4dEnOI to the 4dVar are not clear to me. I would have expected ensemble-based methods like a static 4dEnOI, a EnKF and an ensemble-based smoother. Why a 4dVar that may not use an ensemble? I think that the authors should better motivate this choise.

In response to this comment and Comment M3.3, we added a sentence explaining the choice for 4dVar:

revised text (Section 3.1, par. 3): As a benchmark, we use a strong constraint 4dVar implementation in a 2 outer loop, 10 inner loop setup which was previously presented in Mattern et al. (2017). It uses the log-transformed 4dVar (Song et al. 2012), in which the chlorophyll-a model variable and observations are log-transformed in the computation of the 4dVar cost function and its gradients. We use this 4dVar technique as a benchmark here, because we have used it in the aforementioned studies and are familiar with its configuration.

(S3.16) comment: p.7, l.260-263 "Although, in the following, we assess [...] largest difference in configuration between the DA two systems": It is not clear from the manuscript what is implemented in the 4dVar, but a second main difference between the two systems may be the modelling of the B matrix: one uses an ensemble for the 4dEnOI and the other a static matrix (I presume). If think that more information on the modelling of **B** in the 4dVar system are required.

The matrix changes from month to month and is otherwise static. Similar to the variable localization, entries for unobserved variables were reduced in magnitude to avoid unrealistic increments. More details can be found in Mattern et al. (2017), which, in response to this comment, point out more explicitly:

revised text (Section 3.1, par. 2): As a benchmark, we use a strong constraint 4dVar implementation in a 2 outer loop, 10 inner loop setup which was previously presented in Mattern et al. (2017) where more details about the DA configuration can be found. In each DA cycle, it uses a static background error covariance matrix (which varies from cycle to cycle, based on the month) in which entries for unobserved variables have been lowered to prevent unrealistic DA updates. It is a log-transformed 4dVar (Song et al. 2012), in which the chlorophyll-*a* model variable and observations are log-transformed in the computation of the 4dVar cost function and its gradients.

(S3.17) comment: p.8, l.282-283 "we focus on the reduction in a cost function based on a weighted model-observation misfit": I am sorry, but this diagnostic does not allow to conclude about the performances of the DA systems. There are too many hyper-parameters in both DA systems. So it is clear that a configuration of them will results in a decrease of the model-observation misfit. The evolution of the errors for the unobserved variables is crucial, notably with biogeochemical models. It is not rare to observe an improvement of the surface phytoplankton concentration while the errors in the nutrient concetrations are increasing over time (with seasonal cycle). The authors must assessed the performances of the systems with observations that have not been assimilated. Some in situ data must be available in this area and must be used for the intercomparison.

It is true that there are numerous hyperparameters in the 4dEnOI, but as pointed out in response to Comment M2.3, not more than in other comparable DA systems like an EnKF. Currently, unobserved variables are an unavoidable fact of biogeochemical DA. And as previously pointed out in response to Comment M3.1, we think that the evaluation of the prior error can be very valuable.

(S3.18) comment: p.9, l309-312 "while the 4dVar DA system creates [...] especially chlorophyll-a": The impact of the modelling of B should be added in the discussion regarding the 4dVar system. We note on figure 4 that the NO_3 component of the increment is really low, implying no correction on this variable. The authors provide the information latter in the text.

In response, we now mention this earlier in the manuscript, see our response to Comment S3.16.

(S3.19) comment: p.9, l.320 "the 4dVar increments created via tangent linear and adjoint dynamics": Again, the modelling of B can have a strong impact on the increments. It should be mentioned here as well.

We agree and added "and the choice of background error covariance values" to the sentence in question.

(S3.20) comment: p.9 l.336 - p.10 l.339 "the lower prior error values 4dEnOI [...] beneficial dynamical feedback into the next DA cycles": That's not true. Because the diagnostics are computed from the observed variables only, we can not guess what happens for the unobserved, especially the nutrient. Depending of the configuration of the DA systems, it is possible to control the dynamics of the errors in the observed variables, while having a damage of the unobserved variables. That's why the authors must compute diagnostics with additional data.

As we mention above, it is true that there are unobserved variables for which we cannot verify if the increment was beneficial – this is mentioned in the manuscript and the readers are aware of it. What we allude to in this passage is that unrealistic increments to an unobserved variable can feed back to observed variables. For example, a large injection of nutrients created by the DA system may not be immediately noticeable in the current cycle, but could lead to unrealistic phytoplankton levels in the time following the cycle, and would result in an increase in the prior error. We do not notice such deleterious feedback to an observed variable, which makes a bad increment less likely.

(S3.21) comment: p.10, l.362-363 "In our experiments, we use relatively strong variable localization with lvar = 0.1 when variable localization is applied": So basically, the best set up of the experiments is obtained when the cross-corellation among biogeochemical variables are removed. Waht the point to apply multivariate analysis schemes if ones needs to apply trick to make them monovariate? What does highlight these values is that the covariance matrix estimated from the ensemble is not relevant. Rather than trying to stick to the observations, I would rather try to improve the strategy to estimate the forecast error covariance matrix in an EnOI framework, or migrate to nonlinear updates.

As we now state in the manuscript, we only tested values of $l_{\text{var}} = 1, 0.3, 0.1$, and 0.03 of which 0.1 performed best for physical-biogeochemical and biogeochemical-biogeochemical correlations (see reply to Comment S3.13). So, some correlation performed better than none (or $l_{\text{var}} = 0.03$), but we agree that $l_{\text{var}} = 0.1$ represents a strong reduction. We also show the results without variable localization, which are slightly worse, and mention that a more sophisticated variable localization approaches may yield better results with less strong localization, depending on the dynamical distance between the variables (see reply to Comment S3.12). Spurious correlations in small ensembles and unobserved variables in biogeochemical models exist; we do not think this is a reason to give up on biogeochemical DA. Finally, as stated above, adjustment of one observed variable in biogeochemical models does influence many other variables through the forward model. Unlike ocean physics, in which state variables are quite independent (e.g., there is no mass transfer from temperature to salinity), biogeochemical models routinely cycle their fundamental currency (e.g., nitrogen) between state vector elements. Thus, adjustments of phytoplankton, through observations of surface chlorophyll, adjust total nitrogen in the system and directly impact zooplankton, dissolved and particulate matter, and inorganic nitrogen in subsequent cycles through the forward model.

(S3.22) comment: p.10, l.365-366 "With variable localization applied to all variables in L1, the prior and posterior errors increase by a few percent from the 4dEnOI reference": Could the authors explain why the performances of the DA system are degraded in the physical components with this variable localization strategy? To my point, adding hyperparameters that are tuned offline without a clear statistical rational, to compensate for sampling errors in the forecast error covariance matrix or nonlinera correlations among variables, does not seem to be relevant, neither in an EnKF nor an EnOI framework. I wonder what are the performances of a system with lvar = 0 (no cross-corellation among biogeochemical variables)? Would it be as good as the reference?

This comment harks back to some of the previous comments. We now mention how l_{var} was selected, based on a series of DA experiments. Again, l_{var} is only applied to biogeochemical-biogeochemical and physical-biogeochemical correlations, a value of $l_{\text{var}} = 0.03$ performed worse than 0.1, but, yes, the update to unobserved variables remains low. Applying variable localization to physical-physical correlations has a negative effect on SLA results:

(Section 3.4, par. 2): With variable localization applied to all variables in L_1, the prior and posterior errors increase by a few percent from the 4dEnOI reference (Fig 3b). Proportionally, the largest increase in the cost function occurs for SLA observations, highlighting the importance of adjustments of temperature and salinity (and hence interior density) to correct sea surface height. Thus, adding the same level of variable localization that is found to be beneficial for physical-biogeochemical covariances to the SLA-temperature and SLA-salinity covariances has a (modestly) detrimental impact.

(S3.23) comment: p.11, l.399-400 "by reducing the error in the matrix inversion": In an EnOI framweork, there is no reasons to inverse a matrix, because ones needs to solve only one linear system. The authors may consider to reword the sentence.

Addressed in response to Comment S3.11.

(S3.24) comment: p.12, l.419-426 "In most cycles, the second increment further derceases the cost function, but in some cycles the cost function increases [...] in the model-observation difference $\mathbf{y} - \mathbf{Hx}$." Could it not be related to the fact that the Gauss-Newon algorithm has not guarantee of global convergence regarding the minimization of the cost function?

Potentially, but here we cannot forget that the first ensemble member and, with it, the ensemble changes in each iteration. So we think it is more likely that the cause is the increased sensitivities to errors in the ensemble-based covariance estimates, as stated in the text quoted above.

(S3.25) comment: p.12, l.446 "or the inverse matrix": Again, it is must cheaper to perform a factorization of the matrix, and solve few systems than computing the inverse of the matrix. Please reword.

Addressed in response to Comment S3.11.

(S3.26) comment: p.12-13 "3.5 Ensemble regeneration": The authors apply a strategy for which a generation of the ensemble is applied for predefined cycles (2 and 3). I wonder why they did not try to detect when it was required (which statistics to detect it?) rather defining a priori cycles. We might expect to note divergence between the ensemble and the updated member after several cycles as well. I think the authors should better motivate this choice.

The main motivation for using an early cycle to regenerate the ensemble is the large improvement in both prior (forecast) and posterior error following the first assimilation cycle. According to the results in Section 3.3, having an ensemble member closer to the observations may yield improved statistics. Thus, we regenerate the ensemble following the first large improvement, and one cycle later to examine if that yielded a large change in results. Because we do not see a large discrepancy between the results of G_1 and G_2, we do not expect large changes when performing the regeneration in a later cycle. In response to this comment, we have modified the manuscript to point this motivation out more explicitly:

revised text (Section 3.6, par. 1): While the first ensemble member is updated with observations, the remaining, static, 4dEnOI ensemble was generated from the first ensemble member before any data is assimilated (see Section 2.2) and remains static, without any further modifications from data. While a static ensemble has computational benefits (the variable localization experiments or the multiple iteration experiments above can be performed without the need to rerun the full ensemble), the results from Section 3.3 indicate that an ensemble that ensemble members closer to the observations can improve the ensemble-based statistics and yield better stat estimates. An obvious approach to keep the remaining ensemble distributed around the first ensemble member which has been updated with previous observations, is to regenerate the ensemble from the first ensemble member after a number of DA cycles have been performed.

(S3.27) comment: p.14, l.547-548 ”a positive effect that is difficult to measure due to the lack of observations for these variables”: I think that’s the main issue. Because this variable inflation, and the way to tune the hyperparemeters, which have no mathematical grounds, it is really unsatisfactory to not see diagnostics on unassimilated variables. That’s why I urge the authors to provide diagnostics with independant data.

We are not sure, what hyperparameters are assumed to have no mathematical grounds. Variable localization is based on a similar reasoning as spatial localization, it reduces the increments to unobserved variables. If anything, removing variable localization will make negative impacts of the DA system more likely, akin to removing spatial localization in order to allow increments to unobserved parts of the model domain. Unfortunately, there is no data for most biogeochemical variables, but fortunately, the prior error can be a useful metric besides this, see our reply to Comment M3.1.

(S3.28) comment: p.14, l.554-560 ”Similarly, we tested a few variables localization configurations [...] compared to nutrient-zooplankton covariances”: This affirmation is somehow speculative and unfortunately the authors do not provide strategies to compute the localization coefficients. It seems to improve the results for this specific configuration, based on statistics built on the assimilated observations. So it is not clear if the unassimilated variables are improved, and at which extend this approach can be generalized.

We have addressed this comment in our replies to Comment M3.1 and M2.4.

(S3.29) comment: p.15, l.576-577 ”We note that an EnKF update in our implementation would still be computationally more costly [...] rather than a single model state”: This is true, but considering that the forecast step is the most expensive, the regeneration of the ensemble requires a SVD (or a subspace method for eigenvalue), and finally that the EnOI approach does not update the ensemble anomaly, I wonder at which point an EnKF with the same ensemble size remains a good alternative. Unfortunately, there is no comparisons to an EnKF in the numerical experiments.

In response to this and multiple comments above, we have modified the description of the technique, see the note at the top of this document. The reason for not including an EnKF is provided in our reply to Comment M3.3.

(S3.30) comment: p.15, l. 581-583 ”because it requires the inversion of a $n_{obs} \times n_{obs}$” matrix. Numerically, more efficient techniques to compute or approximate this matrix inversion, werre not investigated in this study”: Because there is only one system to solve in the EnOI framework, the cheapest and most robust strategy to use is simply to solve the system: Cholesky factorization, preconditioned conjugate gradients.

Addressed in response to Comment S3.11.

References

J. L. Anderson. Exploring the need for localization in ensemble data assimilation using a hierarchical ensemble filter. *Physica D: Nonlinear Phenomena*, 230(1-2):99–111, 2007. doi: 10.1016/j.physd.2006.02.011.

J. L. Anderson and N. Collins. Scalable implementations of ensemble filter algorithms for data assimilation. *Journal of Atmospheric and Oceanic Technology*, 24(8):1452–1463, 2007. doi: 10.1175/JTECH2049.1.

A. Barth, A. Alvera-Azcárate, and R. H. Weisberg. Assimilation of high-frequency radar currents in a nested model of the West Florida Shelf. *Journal of Geophysical Research: Oceans*, 113(8):1–15, 2008. doi: 10.1029/2007JC004585.

J. Blyverket, P. D. Hamer, L. Bertino, C. Albergel, D. Fairbairn, and W. A. Lahoz. An evaluation of the EnKF vs. EnOI and the assimilation of SMAP, SMOS and ESA CCI soil moisture data over the contiguous US. *Remote Sensing*, 11(5), 2019. doi: 10.3390/rs11050478.

F. Counillon and L. Bertino. Ensemble Optimal Interpolation: multivariate properties in the Gulf of Mexico. *Tellus A: Dynamic Meteorology and Oceanography*, 61(2):296–308, 2009. doi: 10.1111/j.1600-0870.2008.00383.x.

H. J. Hendricks Franssen and W. Kinzelbach. Real-time groundwater flow modeling with the Ensemble Kalman Filter: Joint estimation of states and parameters and the filter inbreeding problem. *Water Resources Research*, 44(9):1–21, 2008. doi: 10.1029/2007WR006505.

P. L. Houtekamer and H. L. Mitchell. A sequential ensemble Kalman filter for atmospheric data assimilation. *Monthly Weather Review*, 129(1):123–137, 2001. doi: 10.1175/1520-0493(2001)129¡0123:ASEKFF¿2.0.CO;2.

J. Hu, K. Fennel, J. P. Mattern, and J. Wilkin. Data assimilation with a local Ensemble Kalman Filter applied to a three-dimensional biological model of the Middle Atlantic Bight. *Journal of Marine Systems*, 94:145–156, 2012. doi: 10.1016/j.jmarsys.2011.11.016.

F. Kang, J. Li, and Z. Ma. Rosenbrock artificial bee colony algorithm for accurate global optimization of numerical functions. *Information Sciences*, 181(16):3508–3531, 2011. doi: 10.1016/j.ins.2011.04.024.

J. P. Mattern and C. A. Edwards. Simple parameter estimation for complex models — Testing evolutionary techniques on 3-dimensional biogeochemical ocean models. *Journal of Marine Systems*, 165:139–152, 2017. doi: 10.1016/j.jmarsys.2016.10.012.

J. P. Mattern, H. Song, C. A. Edwards, A. M. Moore, and J. Fiechter. Data assimilation of physical and chlorophyll a observations in the California Current System using two biogeochemical models. *Ocean Modelling*, 109:55–71, 2017. doi: 10.1016/j.ocemod.2016.12.002.

A. Moore, J. Zavala-Garay, H. G. Arango, C. A. Edwards, J. Anderson, and T. Hoar. Regional and basin scale applications of ensemble adjustment Kalman filter and 4D-Var ocean data assimilation systems. *Progress in Oceanography*, 189(September):102450, 2020. doi: 10.1016/j.pocean.2020.102450.

C. Pan, L. Zheng, R. H. Weisberg, Y. Liu, and C. E. Lembke. Comparisons of different ensemble schemes for glider data assimilation on West Florida Shelf. *Ocean Modelling*, 81:13–24, 2014. doi: 10.1016/j.ocemod.2014.06.005.

P. Sakov, F. Counillon, L. Bertino, K. A. Lister, P. R. Oke, and A. Korablev. TOPAZ4: An ocean-sea ice data assimilation system for the North Atlantic and Arctic. *Ocean Science*, 8(4):633–656, 2012. doi: 10.5194/os-8-633-2012.

H. Song, C. A. Edwards, A. M. Moore, and J. Fiechter. Incremental four-dimensional variational data assimilation of positive-definite oceanic variables using a logarithm transformation. *Ocean Modelling*, 54-55:1–17, 2012. doi: 10.1016/j.ocemod.2012.06.001.

V. Vervatis, C. E. Testut, P. De Mey, N. Ayoub, J. Chanut, and G. Quattrocchi. Data assimilative twin-experiment in a high-resolution Bay of Biscay configuration: 4DEnOI based on stochastic modeling of the wind forcing. *Ocean Modelling*, 100:1–19, 2016. doi: 10.1016/j.ocemod.2016.01.003.

X. Wang and T. Lei. GSI-based four-dimensional ensemble-variational (4DEnsVar) data assimilation: Formulation and single-resolution experiments with real data for NCEP global forecast system. *Monthly Weather Review*, 142(9):3303–3325, 2014. doi: 10.1175/MWR-D-13-00303.1.

M. Yaremchuk, P. Martin, and C. Beattie. A hybrid approach to generating search subspaces in dynamically constrained 4-dimensional data assimilation. *Ocean Modelling*, 117:41–51, 2017. doi: 10.1016/j.ocemod.2017.08.003.
