# Supplementary material for: Ensemble optimal interpolation for adjoint-free biogeochemical data assimilation
Source: PLoS One. 2023 Sep 5;18(9):e0291039. doi: 10.1371/journal.pone.0291039 (PMC10479889; doi:10.1371/journal.pone.0291039)
Supplement: Attachment — Submitted filename: response_letter_r2.pdf [file pone.0291039.s004.pdf]

Comments of reviewer 1 and responses

major comments

comment: Thank the authors for their efforts to address previous comments. The manuscript has been significantly improved. I have a few comments as below.

We thank the reviewer for the thoughtful comments, please see our responses below.

(M1.1) comment: Since the spatial localization function (6) does not go to zero exactly, any threshold is applied to force it being zero at a certain distance? If not, the spatial localization cannot help with computational efficiency.

Indeed, the spatial localization function does not go to zero, and currently no threshold is applied to force it to zero – these circumstances do not help with computational efficiency, but they do not make the procedure much more inefficient either. First off, we apply localization to the relatively small matrix $\mathbf{HBH}^{\mathrm{T}}$ once per DA update. For the larger $\mathbf{BH}^{\mathrm{T}}$ which is computed in parts ("slices" in S1), the weights (entries of $\mathbf{L}$) are pre-computed separately in x- and y-direction as they can be re-applied to every tracer variable. We performed simply a point-wise multiplication of the pre-computed weights with each slice of $\mathbf{BH}^{\mathrm{T}}$ as it is being processed. This procedure is relatively efficient, but it is possible that additional efficiency can be gained by carefully indexing only those weights above a certain threshold, and by avoiding the computation of some (near-zero) covariance terms altogether. In response, we augmented the appendix with this information:

revised text (Section S1, par. 3): Here, we did not examine an additional step to improve the computational efficiency of the DA update step, which is referred to as "local analysis" (Sakov and Bertino 2011) or "domain localization" (Carrassi et al. 2018): By applying a threshold to the localization weights and setting entries in $\mathbf{L}$ that are below the threshold to zero, spatially distant model state and observation locations can be ignored in the computation of $\mathbf{BH}^{\mathrm{T}}$. For example, when processing a slice of the model state associated with a given horizontal coordinate, spatially distant observations, whose localization weights are zero for the whole slice, can be ignored, setting entries in $\mathbf{s}[i_k : i_{k+1}]$ to zero, without even computing the covariance term in Eq. (S2).

(M1.2) comment: The authors replied that it is hard to explain why 4DEnOI outperforms 4DVAR. But it would be nice to have some discussions or thoughts on this counterintuitive result. Is it possible that the static B at the initial time for 4DVAR is not as good as that in 4DEnOI, or the propagation of B during an assimilation window is harmful for some reasons?

Like the reviewer, we suspect that the static $\mathbf{B}$ is likely to blame for comparatively bad results of the 4DVar technique in our results. Firstly, it is difficult to determine suitable values for the entries of $\mathbf{B}$ which we have examined previously (Mattern et al. 2018). Secondly, and perhaps more importantly here, ROMS only permits users to set the diagonal entries of $\mathbf{B}$ (which are extended to off-diagonal elements associated with the same variable using length scales based on a solution to a diffusion problem), so that covariance terms between different variables are set to zero. As a result, increments to unobserved variables are related to increments to observed variables through the tangent-linear and adjoint dynamics but not considered in the cost function. In previous experiments, this circumstance led to sometimes strange increments. In an experiment in Mattern et al. (2017), we examined a steady increase of surface NO_3 to unrealistically high levels in the northern part of the model domain across several DA cycles. This increase was caused by the 4dVar DA, which aimed to increase surface phytoplankton/chlorophyll-a. Because phytoplankton growth was largely light-limited in that location, a steady increase in NO_3 only caused a minor improvement in model observation misfit. Meanwhile, the increment was not sufficiently penalized by the entries of $\mathbf{B}$, and without covariance terms linking phytoplankton and NO_3, we were required to down-weight the diagonal elements of $\mathbf{B}$ associated with the NO_3 variable. Following the

reviewer's comment and Comment M1.2, we discuss these drawbacks of the 4dVar implementation in Section 4.

Comments of reviewer 4 and responses

comment: The manuscript describes the application of an ensemble-like approach for coupled biogeochemical and physical data assimilation in marine environments. The ensemble used in the assimilation is composed of a static part (like in EnOI) with the addition of the assimilated trajectory-. If necessary, the static part of the ensemble can be regenerated. Results of the application of the proposed assimilation method (named 4dEnOI) for a relatively simple biogeochemical model have been compared with a previously existing 4dVar. Moreover, different settings of 4dEnOI are presented to investigate the effects of a number of elements of the assimilation method (e.g., localization, ensemble regeneration, inclusion of the assimilated trajectory in the ensemble, and iteration of the EnOI).

We thank the reviewer for the thoughtful comments, please see our responses below.

... The manuscript is well written and changes in the revised manuscript try to respond to all the comments and suggestions raised in the previous review round. However, in my opinion the manuscript can be further improved considering its main strong and weak points.
The main strong points that I see are:

major comments

(M4.1) comment: The method proposed is a two-way strongly coupled biogeochemical-physical assimilation. Even if variable localization is applied, and thus the coupling between physical and biogeochemical variables is weakened, this is not a trivial task. In addition, the variable localization is one of the aspects investigated in the manuscript and the results show possible negative effects of strong coupling of all the physical and biogeochemical variables. In my opinion this aspect should be better highlighted in the Discussion & conclusions and in the abstract.

We agree with the reviewer, and in response, we added a few sentences to the abstract and the Discussion & conclusion section, with the goal of emphasizing the role of variable localization and the interpretation of its results:

revised text (Abstract): We show that the 4dEnOI results depend heavily on covariance localization generally, and benefit from variable localization in particular, when it is applied to reduce the coupling strength between the physical and biogeochemical model and the biogeochemical variables.

revised text (Section 4, par. 4): We also tested a simple variable localization scheme which, when combined with the spatial localization, provides the best results in this study. That is, retaining the strong coupling between the physical model variables, while reducing physical-biogeochemical and biogeochemical-biogeochemical covariance terms yielded the lowest model-observation misfit in our experiments. This result suggest that the correlations of less related – dynamically distant – variables are overestimated by the ensemble, analogous to correlations between spatially distant grid points. Beyond decreasing the model-observation misfit, variable localization likely also reduces unrealistic increments to unobserved biogeochemical variables, a positive effect that is difficult to measure due to the lack of observations for these variables.

... Consider to clearly highlight also in the introduction that both physical and biogeochemical variables are assimilated in a coupled DA method.

We followed the reviewer's suggestion and highlight this aspect more strongly in the introduction:

revised text (Section 1, par. 4): With the purpose of assimilating physical and biogeochemical observations jointly into a coupled physical-biogeochemical model, we implement a version of EnOI, that retains one of the main characteristics of the EnOI presented in Evensen (2003): [...]

(M4.2) comment: The manuscript proposes a method that has performances similar to the 4dVar and that does not require tangent linear or adjoint code. This point is already well declared in the Discussion & conclusions. This point could be further analyzed in the framework of operational applications. Is the assimilation method suitable to be implemented in an operational system? Is it more suitable than 4dVar?

We agree with the reviewer that such an analysis could be of interest to the readers. In response, we modified the last two paragraphs of Section 4, to add our thoughts about using this 4dEnOI implementation in an operational setting. In short, we recommend its use for models for which no tangent-linear and adjoint models exist and are difficult to create, and we emphasize its utility for properly calibrating localization length scales and similar parameters used in ensemble-based DA without the need to run a full ensemble.

comment: On the other hand the revised manuscript is still affected by some weaknesses:

(M4.3) comment: The 4dEnOI is applied using a relatively simple biogeochemical model. Nothing against this choice but in the abstract and in the conclusions the results are presented citing largely more complex biogeochemical models (first two row of the abstract; and L. 541-543). I suggest to add some discussion about the limitations of using a "simple" NPDZ model and if there are elements that suggest that the results presented in the manuscript can be extended to complex biogeochemical models.

We agree with the reviewer's point. Assimilating data into a more complex biogeochemical model does indeed pose challenges that are not as pronounced in simpler models, notably the large number of unobserved variables and a weaker average linkage strength between variables. In response, we added a brief discussion of this aspect to Section 4 par. 6 which further includes a discussion based on Comment M1.2.

(M4.4) comment: The number of ensemble members is fixed to 25. Why this number of ensemble members? Do you expect that results will be different with a larger number of ensemble members? Maybe the fact of including the assimilated trajectory in the ensemble would be less relevant and the performances would be more similar to the ones of an EnOI, if a larger ensemble was adopted.

The choice of using 25 ensemble members in all of our experiments was mainly based on the cluster computer available to us. Furthermore, we already examine many aspects of the 4dEnOI implementation, and did not want to extend the list of experiments (and the results section) by showing the effect of using different ensemble sizes.

We agree with the reviewer that a larger ensemble would likely diminish the importance of including the first ensemble member in the ensemble statistics. In response, we have added this information to the discussion section:

revised text (Section 4, par. 3): In our experiments, the choice of 25 ensemble members was mainly based on the computing resources available to us. We presume that a larger ensemble would improve the 4dEnOI results, while also reducing the relevance of including the first, and only non-static, ensemble member in the comutation of the ensemble statistics (see Section 3.2).

(M4.5) comment: The manuscript shows that the assimilation performances benefit from including the assimilated trajectory in the ensemble, however, similarly to the other Reviewers, I am not fully convinced about the mathematical consistency of the approach. Indeed, the ensemble that includes x1 has been not generated by a statistically consistent formulation. Maybe I am missing some points but I suggest the Authors to place 4dEnOI in the framework of the very comprehensive review by Carrassi et al., (2018) and to discuss possible limitations or drawbacks of the methods related to the fact that only one of the ensemble member is updated.

Carrassi et al. (2018) provides a rigorous review and description of DA in the geosciences. While EnOI with static ensembles are not mentioned explicitly, the paper describes and discusses a few DA methods that hybridize a static error covariance matrix with a dynamical one sampled from an ensemble. While these hybrid methods typically refer to complex ensemble-variational DA techniques, the 4dEnOI technique presented here performs a similar but perhaps simpler hybridization of the covariances: it is generated from a static ensemble augmented by a (single-member) dynamical one. As such, we do not see a mathematical inconsistency in the formulation of the 4dEnOI technique. A drawback of the technique, of course, is that the ensemble is dominated by static members, and, as we have shown, is improved by including a dynamical ensemble member. Including more dynamical members would bring the technique closer to an EnKF, which might yet yield better results.

In response to this comment, we now explicitly mention the hybrid nature of background error covariance matrix and reference a paper using a similar approach applied to the EnKF:

revised text (Section 4, par. 2): The resulting technique thus creates a hybrid background error covariance matrix generated from a hybrid ensemble – a mostly static ensemble augmented by a (single-member) dynamical one – analogous to EnKF implementations that use hybrid covariances (see, e.g., Counillon et al. 2009).

(M4.6) comment: After the first revision the manuscript has been modified to show that, even if the ensemble was not really static in the present applications, results would have been the same with a static ensemble (leaving apart x1). In my opinion, this point could be not fully understandable for the readers, therefore I suggest presenting the method as if the ensemble (from x2 to xn) is static in all the simulations except for the ones including regeneration. This approach requires changes in different parts of the manuscript but, in my opinion, it can considerably improve the presentation of 4dEnOI.

We agree with the reviewer that this approach is beneficial to readers. In response, we went through the manuscript carefully and reworded sections where needed.

specific comments

(S4.1) comment: L. 44-53 The text from "primarily construction" to "easy to implement" seems redundant and out of the main topic of the manuscript, since details are provided in Mattern et al. (2019). I suggest removing (or at least strongly reducing) this part.

We agree with the reviewer and have removed several sentences from the paragraph in question.

(S4.2) comment: L. 56-57 The EnKF is defined as the "best" among the ensemble-based DA techniques. Usually a DA approach can be better than others depending on the application, on the settings, on the model complexity. Thus, the claim that the EnKF is the "best" should be motivated considering the different available ensemble-based DA techniques and referring to relevant references.

This comment is based on a misunderstanding. We refer to the EnKF as the "best known" in that sentence. We do not imply – and do not mean to imply – that the EnKF is the best-performing ensemble-based DA technique.

(S4.3) comment: L. 73 Change "(Verdy et al. 2016)" in "Verdy et al. (2016)".

We corrected the formatting of the reference. To adhere to PLOS formatting rules, all citations are now numerical.

(S4.4) comment: L. 74 "included in the computation" ("in" is missing).

Corrected.

(S4.5) comment: L. 112 I suggest writing that observation locations are intended as spatial and temporal locations.

We agree with the reviewer, and now explicitly refer to "spatial and temporal observation locations" in that sentence.

(S4.6) comment: In the Fig. 2 caption, it should be added also regeneration: "the initial model state (ensemble generation) or a successive model state (ensemble regeneration) is used as the reference solution".

Following the reviewer's suggestion, we modified the figure caption. It now reads:

revised text: Model snapshots are obtained from daily snapshots of a 5-year-long, non-assimilative model simulation; the initial model state (ensemble generation) or a successive model state (ensemble regeneration) is used as the reference solution from which the ensemble is created.

(S4.7) comment: L. 186 I suggest changing "localization" into "spatial localization".

We agree, and added the word.

(S4.8) comment: In my opinion section 3.1 should go in the Methods section.

We agree with the reviewer, and moved most of former Section 3.1 to the new Section 2.5, which is part of the Methods section.

... Moreover, observation errors are not presented in section 3.1 but it would be relevant to know how they are treated in 4dEnOI (and in 4dvar).

In response to this comment, the latter part of former Section 3.1 has now been moved to a new "Observations" section (Section 2.6). This new section contains information about the observation types, the observation processing ("super-obbing"), the observation error values used in this study, and the spatial distribution of the observations for each type that was assimilated.

(S4.9) comment: L. 264 Change "(Veneziani et al. 2009)" in "Veneziani et al. (2009)".

Corrected; to adhere to PLOS formatting rules, all citations are now numerical.

(S4.10) comment: In the last two rows of Table 1 chlorophyll glider observations are listed. Are these observations assimilated or used for validation?

All observations in the table are used in the assimilation as stated in the caption. In this study, we did not examine the effects of the assimilation on non-assimilated observations and focused on the comparison between 4dVar and 4dEnOI. We examined the improvement for non-assimilated observations in a previous study for the

4dVar-based DA system (Mattern et al. 2017); in a future study, we hope to include similar metrics for a more complex biogeochemical model.

(S4.11) comment: A number of abbreviations are used in the headers of Table 2. I suggest explaing them in the caption.

We agree with the reviewer that the abbreviations need to be explained. Instead of including descriptions in the caption, we opted to use footnotes below the table for that purpose.

(S4.12) comment: In the Fig. 3 caption and at L. 309, I think that "normalized Jobs (eq. 7)" should be used instead of "cost function".

After reading the caption of Fig 4 (former Fig. 3) and the associated manuscript section, we noticed that we use the term "cost function" widely when referring to J_{obs}. We prefer the use of "cost function" simply because it is not a mathematical symbol appearing in the text. Yet, we agree with the reviewer that this notation needs to be introduced when used initially. In response to this comment, we added a remark preceding the first use of "cost function" as J_{obs}:

revised text (Section 3.1, par. 1): While 4dVar and 4dEnOI thus optimize different cost functions, the main contributor to both cost functions is J_{obs}, which we use here as a metric of evaluation and which we hereafter refer to as cost function.

In the caption of Fig 4, which has three mentions of "cost function", we further added a reference to J_{obs} and Eq. (7), as suggested by the reviewer.

(S4.13) comment: L. 333 Commenting Fig. 4 the Authors refer to "chlorophyll-a" while in Fig. 4 "phytoplankton" is used.

Good catch, we now use "phytoplankton" in the text, because it is directly referring to the model variable and not observations.

(S4.14) comment: Some patterns appear in the chlorophyll (phytoplankton increment) In Fig. 4. Are the satellite based chlorophyll observations affected by cloud coverage? If yes, are increments larger where observations are available? Cloud coverage and more generally some details about the temporal and spatial distribution of observations is missing (not only for chlorophyll).

Yes, the satellite-based chlorophyll-*a* observations are affected by cloud coverage and, yes, increments tend to be larger in locations where observations are available, due to the horizontal length scales employed in the DA techniques. However, there are other important factors affecting the size of the DA increment, which also have spatial dependence: model-observation misfit, observation error and DA background error. Thus, it is not easy to attribute the size of an increment to a single factor.

In response to the reviewer's comment and Comment S4.8, we have added Section 2.6 to the manuscript. This section includes the new Fig 3, showing the spatial distribution and average frequency for all observation types that were assimilated, using 2-dimensional histograms. While individual grid cells closer to the coast are observed slightly more frequently than those offshore (Fig 3a), there is little evidence that a bias in the spatial chlorophyll-*a* satellite data distribution is a leading cause for larger increments close to the coast.

(S4.15) comment: L. 381-386 Fig. 5 shows that leaving out the x1 member the correlation of the ensemble increases more than leaving out any other ensemble member. Is this an intrinsic result of the fact that, being x1 corrected by DA, it is less correlated to the other static ensemble members? I suggest considering this aspect when commenting Fig. 5.

Indeed, the reviewer is correct, we computed the correlations between the different ensemble members, which

showed a lower correlation between the first ensemble member and the remaining ensemble. In response, we have now included this information in the manuscript:

revised text (Section 3.2, par. 2): The examination further reveals that the first ensemble member is less correlated to the static ensemble members than these are among themselves (not shown).

(S4.16) comment: L. 388 I am not sure that "improved ensemble statistics" is correct. Indeed, it is not demonstrated that the mean or the covariance of the ensemble including x1 are better than those of the purely static ensemble. What the Authors can infer is based on normalized Jobs (ensemble performances).

We agree with the reviewer that we should be more careful with the wording here. In response, we modified the sentence in question; it now reads:

revised text (Section 3.2, par. 2): Thus, including a non-static ensemble member, that has been updated with observations, appears to provide noticeably different ensemble statistics for the 4dEnOI updates, which improve the fit to the assimilated observations in our experiments.

References

A. Carrassi, M. Bocquet, L. Bertino, and G. Evensen. Data assimilation in the geosciences: An overview of methods, issues, and perspectives. *Wiley Interdisciplinary Reviews: Climate Change*, 9(5):1–50, 2018. doi: 10.1002/wcc.535.

F. Counillon, P. Sakov, and L. Bertino. Application of a hybrid EnKF-OI to ocean forecasting. *Ocean Science*, 5(4):389–401, 2009. doi: 10.5194/os-5-389-2009.

G. Evensen. The Ensemble Kalman Filter: theoretical formulation and practical implementation. *Ocean Dynamics*, 53(4):343–367, 2003. doi: 10.1007/s10236-003-0036-9.

J. P. Mattern, H. Song, C. A. Edwards, A. M. Moore, and J. Fiechter. Data assimilation of physical and chlorophyll a observations in the California Current System using two biogeochemical models. *Ocean Modelling*, 109:55–71, 2017. doi: 10.1016/j.ocemod.2016.12.002.

J. P. Mattern, C. A. Edwards, and A. M. Moore. Improving Variational Data Assimilation through Background and Observation Error Adjustments. *Monthly Weather Review*, 146(2):485–501, 2018. doi: 10.1175/MWR-D-17-0263.1.

P. Sakov and L. Bertino. Relation between two common localisation methods for the EnKF. *Computational Geosciences*, 15(2):225–237, 2011. doi: 10.1007/s10596-010-9202-6.
